# *Diphyllobothrium latum* in a Russian Citizen Traveling in China

**DOI:** 10.4269/ajtmh.23-0335

**Published:** 2023-08-07

**Authors:** Bingchao Bao, Jinlin Liu, Wei Pan

**Affiliations:** ^1^ Department of Clinical Laboratory, Longgang District People’s Hospital of Shenzhen & The Second Affiliated Hospital of the Chinese University of Hong Kong, Shenzhen, China;; ^2^ Department of Clinical Laboratory, South China Hospital, Medical School, Shenzhen University, Shenzhen, China;; ^3^ Department of Clinical Laboratory, Haiyan People’s Hospital, Haiyan, China

A 59-year-old male Russian traveling in China was admitted to our hospital for a physical examination and capsule endoscopy. During the previous year, he did not complain of abdominal pain or other health issues. His laboratory test result was unremarkable, with no evidence of anemia (hemoglobin 15.0 g/dL). Capsule endoscopy revealed a 90 cm long, yellow rose-like tapeworm, *Diphyllobothrium latum*, in his duodenum, extending into the ileum (Figure 1A and B and Supplemental Video). A careful reexamination of the stool revealed no eggs. Subsequently, a fragment of *D. latum* pressed between two microscopic slides and observed under a microscope (400×) (Figure 1C) revealed numerous eggs. *D. latum* is a fish tapeworm that can infect humans after consumption of infected undercooked or raw fish.^1^^,^^2^ The patient had a history of consuming barracuda eggs 1 year before and was treated with a single dose of praziquantel.

**Figure 1.**
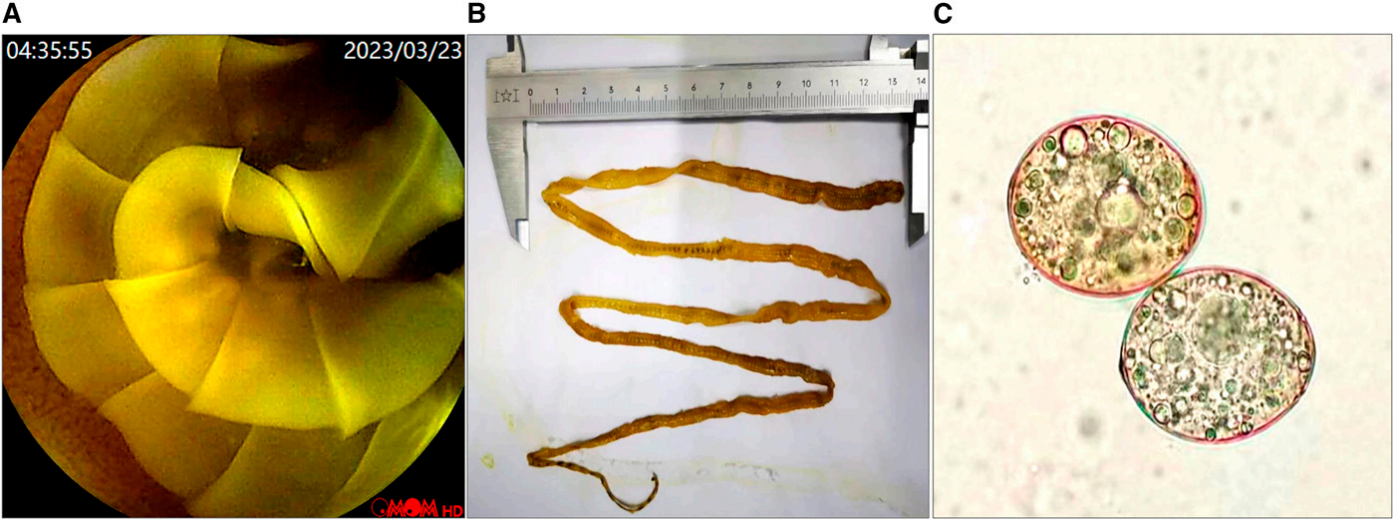
Russian traveler with *Diphyllobothrium latum*. Morphological characterization of *D. latum* using capsule endoscopy (**A**), general view (**B**), and the eggs under microscopy (**C**).

Here, we present a case of intestinal *D. latum* (also called the “fish tapeworm” or the “broad tapeworm”), an emerging zoonosis, as well as its morphological and general view using capsule endoscopy and microscopic examination of its eggs. *D. latum* is transmitted to humans through the consumption of fish containing the infectious larvae. Moreover, this case demonstrates that patients infected with *D. latum* could be asymptomatic and the importance of regular medical checkups. However, if the worm burden is high, the patient could present with abdominal pain, discomfort, diarrhea, and malaise.^3^^,^^4^ Avoiding raw fish and eggs is the most effective way to prevent *D. latum* infection, thus preventing complications if the worm burden is high.^5^

## Supplemental Materials


Supplemental materials
